# Antidepressants amitriptyline, fluoxetine, and traditional Chinese medicine Xiaoyaosan caused alterations in gut DNA virome composition and function in rats exposed chronic unpredictable mild stress

**DOI:** 10.3389/fmicb.2023.1132403

**Published:** 2023-04-14

**Authors:** Jialin Li, Wan Qu, Chengcheng Hu, Zongbao Liu, He Yan

**Affiliations:** ^1^School of Food Science and Engineering, South China University of Technology, Guangzhou, China; ^2^Nanyang Technological University Food Technology Centre (NAFTEC), Nanyang Technological University (NTU), Singapore, Singapore; ^3^Laboratory of Ecology of Rare and Endangered Species and Environmental Protection (Guangxi Normal University), Ministry of Education, Guilin, Guangxi, China; ^4^Guangdong Province Key Laboratory for Green Processing of Natural Products and Product Safety, Guangzhou, China

**Keywords:** gut viruses, depression, amitriptyline, fluoxetine, Xiaoyaosan

## Abstract

**Background:**

In clinical practice, antidepressant drugs are widely used to treat depression. Previous studies have attention to the impact of antidepressants on the bacterial microbiome, while the role of these drugs in the gut virome is still unclear.

**Methods:**

In this study, we estimated the effects of antidepressant amitriptyline (Ami), fluoxetine (Flu), and traditional Chinese medicine Xiaoyaosan (XYS) administration on gut viral composition and function in a chronic unpredictable mild stress (CUMS)-induced depression rat model based on shotgun metagenomic sequencing.

**Results:**

The results showed that treatment with Ami, Flu, and XYS significantly changed the gut viral composition compared with the CUMS-induced rats. At the family level, the abundance of *f_unclassified_Caudovirales* in CUMS rats was remarkably lower than in the HC rats, nevertheless, XYS significantly recovered the abundance of Caudovirales. Meanwhile, the abundance of *Podoviridae* was expanded in CUMS rats compared with the HC rats, and the profile was then significantly reduced after XYS treatment. Furthermore, both antidepressants and XYS increased the abundance of *Siphoviridae* compared with the CUMS rats, but only Ami treatments had significant differences. Subsequent function annotation further implied that Ami, Flu, and XYS showed to involve an alteration of the diverse viral functions, such as carbohydrate metabolism, xenobiotics biodegradation and metabolism, community-prokaryotes, translation, and neurodegenerative disease. Additionally, the co-occurrence network displayed that there are complex interactions between viral operational taxonomic units (vOTUs) represented by temperate phages and the majority of bacterial genera in the intestine ecosystem.

**Conclusion:**

Our study proved for the first time that depression is characterized by massive alterations and functional distortion of the gut viruses, and after oral administration of Ami, Flu, and XYS could affect disordered gut virome, which could be a novel target in depression.

## 1. Introduction

Since the 21st century, depression has become one of the most severe mental diseases affecting human life, characterized by low sensitivity, sadness, sleeplessness, and loss of interest in enjoyable activities, with high morbidity and mortality rates (Kupfer et al., 2012). According to the World Health Organization, depression afflicts over 350 million people worldwide and costs USD 1 trillion annually in lost productivity globally (World Health Organization (WHO), 2022b). With the COVID-19 pandemic, the global prevalence of anxiety and depression has increased by 25% (World Health Organization (WHO), 2022a). Many traditional pathogeneses mainly involve brain factors, such as regulated neuroendocrine (Stetler and Miller, 2011), neuroimmune (Dowlati et al., 2010), and neurotransmitter systems (Berton and Nestler, 2006). However, because of the complexity of the disease, the etiology of depression is still unclear. The intestinal microbiome comprises several thousand species containing bacteria, viruses (bacteriophages), archaea, and fungi, which greatly sustain human health (Hornig, 2013). Numerous studies in the last several years also proved that the intestinal microbiome produces a marked effect on depression *via* the “microbial-intestine-brain” axis (O’Mahony et al., 2009; Park et al., 2013; Kelly et al., 2016; Zheng et al., 2016; Yu et al., 2017; Qu et al., 2019; Yang H. J. et al., 2020; Zhang et al., 2021).

As an integral part of the intestinal microbiome, the gut viral communities (known as the “virome”) also plays a vital role in keeping human healthy (Cadwell, 2015). They are composed of a great quantity of prokaryotic viruses (mostly bacteriophages) and eukaryotic viruses (Shkoporov and Hill, 2019). Recently, many studies have verified that the alterations of enterovirus are related to inflammatory bowel diseases (Norman et al., 2015; Zuo et al., 2019). Gut viruses might be the immediate cause of disease or indirectly affect the evolution of disease by modulating the structure of the microbiota through the cascade of positive and negative interactions between bacterial communities (Gogokhia et al., 2019; Hsu et al., 2019). In certain diseases with a serious imbalance of intestinal flora, disease-specific changes and disturbances of the gut virome have also been discovered, like type 2 diabetes (Ma et al., 2018), pulmonary arterial hypertension (Kim et al., 2020), stunted (Khan Mirzaei et al., 2020), Parkinson’s disease (Tetz et al., 2018) and so on. However, up to now, only two research have described the association between enteroviruses and depression. One study investigated the features of intestinal bacteriophages and bacteria together with their functional potential in patients with major depressive disorder and healthy controls (Yang H. J. et al., 2020). Another study has previously probed into the relationships between the gut virome and neurotransmitter metabolism in a mouse model of depression (Duan et al., 2022). Both studies emphasize the alteration and maladjustment of gut viral abundance in depression, but acquiring as many viruses as possible is problematic because most current viruses do not match the existing database.

Drugs are extensively applied to treat depression (Cipriani et al., 2018). As emphasized by some previous research, many antidepressant drugs have been proven to play a fundamental role in adjusting the gut microbial composition and structure during treatment for depression (Yang et al., 2017; Getachew et al., 2018). Similarly, in our previous study, treatment of Ami and Flu notably altered the abundance of gut microbiota in rats exposed to chronic unpredictable mild stress (Zhang et al., 2021). Nevertheless, at the same time, the side effects of antidepressants raise concerns (Garattini and Samanin, 1988; Bijlsma et al., 2014). Thus, it has raised increasing concern about the new antidepressants found in natural herbs. Recently, increasing evidence has testified that many commonly used traditional Chinese medicines (TCM) may also induce beneficial alterations in the intestinal flora to ameliorate depressive-like behavior (Cao et al., 2020; Feng et al., 2020). Xiaoyaosan (XYS), as a classic preparation in TCM, was first detected in the Song Dynasty’s (960–1,127)’s “Taiping Huimin and Jijufang.” In China, XYS has treated mental disorders for thousands of years. In recent years, Lv et al. detected that XYS may play an antidepressant role by reducing the abundance of harmful bacteria (such as the genera *Facklamia*), increasing the abundance of beneficial bacteria (especially *Lactobacillus*), and restoring abnormal levels of certain cecal metabolites (Lv et al., 2021). However, these research in this field has concentrated on the effects of drugs on the intestinal flora but neglected that gut viruses are also a critical component of the intestinal microbiome.

Increasingly compelling data have shown that drugs could also affect the composition and function of gut viruses. For instance, using the antiviral drug ribavirin could restore the enteric viral community to the healthy state of Experimental Gulf War Illness (Seth et al., 2019). Furthermore, several studies found that many non-antiviral drugs or active ingredients also modulate gut viral composition and function. Yang et al. reported that Quyushengxin formula (QYSX), known as traditional Chinese medicine, could be utilized to treat ulcerative colitis and adjust disordered intestinal bacteria and bacteriophages (Yang J. et al., 2020). Likewise, Dong et al. questioned the correlations between the regulation of gut virome and bacteriome by green tea polyphenols (GTP). They found that GTP regulates the gut microbiota through the gut phage communities (Dong et al., 2022). Moreover, another new study also showed that Andrographolide (AG), a bioactive component extracted from plants, altered the virome with a significantly decreased abundance of *Siphoviridae* and *Myoviridae*, which are known to be associated with gastrointestinal pathology (Saha et al., 2021). However, at present, no study assessing the impacts of antidepressants Ami and Flu and XYS on the gut viral composition and function under conditions of depressed hosts. This forms the basis of our current research.

In light of this, with the incorporation of metagenomic sequencing and virus sequence recognition algorithms, we examined the variations of the gut viral composition and functions in chronic unpredictable mild stress (CUMS)-induced rats model of depression. Importantly, we tested the hypothesis that the use of Ami, Flu, and XYS change the viral community respectively. In addition, we also explored correlation analyses between gut viruses and their bacterial hosts in the intestinal ecosystem. Our findings have augmented the present comprehension of the link between gut viruses and drug action.

## 2. Methods and materials

### 2.1. Animals, treatments, and collection of fecal samples

All animal experiments in this study were conducted in the animal facility of the animal center of South China Agricultural University. Specific pathogen-free adult male Sprague–Dawley rats, purchased from Guangdong Medical Experimental Animal Center, were adapted to the laboratory environment for one week (12 h light/dark cycle, 25 ± 1°C, and 55–65% relative humidity with unrestricted access to food and water). The experimental process was presented in Supplementary Figure S1A. After acclimatization, the rats were randomly allocated into two groups: healthy group (HC; *N* = 12) and chronic unpredictable mild stress group (CUMS; *N* = 54). The detailed CUMS procedures were listed in our previously published studies (Qu et al., 2019). Briefly, CUMS was induced by eight types of stressors: excessive illumination; cage tilt (45°C, 24 h); wet bedding (24 h); tail suspension (6 min); tail pinch (5 min); food and water deprivation (24 h); bake oven (60°C, 5 min) and flashing light at 150 flash/min for 5 min (Li et al., 2013; Liu et al., 2015). The rats were subjected to randomly select one or two stimuli every day and were not to allowed to repeat the same stimulus for two consecutive days. Each stimulus was guaranteed to be applied to the rats about thirteen times. Overall, the animal experiment lasted 15 weeks. Except for HC rats, all depressed rats received eight different chronic unpredictable mild stimulation for 14 weeks. The body weights of all rats were recorded every week. The CUMS-resistant rats were filtered out by the sucrose preference test (SPT), open field test (OFT), and light/dark test (LDT) at the eight weeks of exposure to CUMS. The remaining rats were administered with Xiaoyaosan (XYS; *N* = 12), amitriptyline (Ami; *N* = 6), and fluoxetine (Flu; *N* = 7) respectively for six weeks by oral administration. Then, after six weeks of treatment with Xiaoyaosan (XYS), amitriptyline (Ami), and fluoxetine (Flu), the depression-like behaviors in rats were evaluated by the SPT, LDT, and FST. Fecal samples from the three individual rats in each group (HC: *N* = 3; CUMS: *N* = 3; Ami: *N* = 3; Flu: *N* = 3; XYS: *N* = 3) were selected for metagenomic analysis at 15 weeks and stored at −80°C for subsequent analysis.

### 2.2. DNA extraction, shotgun metagenomic sequencing

DNA was extracted from fecal samples with the QIAmp DNA Stool Mini Kit (Qiagen, USA). Shotgun metagenomic sequencing of all samples was again performed using Illumina HiSeq 4,000 sequencer in Shanghai Majorbio Bio-pharm Technology Co. Ltd. Sequence data associated with this project have been deposited in the NCBI Short Read Archive database (Accession Number: PRJNA910226). Then, the metagenomic raw reads were adjusted quality using Sickle,^1^ and low-quality bases and reads were removed. Burrows–Wheeler Aligner^2^ compared raw reads with the rat genome to remove the relevant host information. After quality control, the self-written Python script was used for subsequent data processing and analysis. All the clean paired-end reads were *de novo* whole-genome assembled using SPAdes with the following parameters: -meta -k 21,33,55,77 -only-assembler (Nurk et al., 2017). Scaffolds longer than 1,000 bp were retained for downstream analysis.

### 2.3. Identification of viral sequences

To determine confident viral scaffolds, the viral sequence identification standard operating procedure (SOP) with VirSorter2 V.3 from protocols.io^3^ was employed, combining VirSorter2, DRAMv, and CheckV, compliance with standard criteria based on viral and host gene counts, score, hallmark gene counts, and contig length (Guo et al., 2021). Additionally, a viral detection pipeline developed by Nayfach et al. (2021) was used to complement the identification of virus sequences. Briefly, this method used a combination of four viral signatures to identify viral genome fragments: (i) the presence of viral protein families, (ii) the absence of microbial protein families, (iii) the presence of viral nucleotide signatures, and (iv) multiple adjacent genes on the same strand. Metagenomic scaffolds were searched for viral and microbial protein families using hmmsearch against Integrated Microbial Genome/Virus (IMG/VR) and Pfam-A database, respectively (Nayfach et al., 2021). VirFinder v.1.1 was applied to evaluate the presence of viral nucleotide signatures. Detailed procedures for selecting viral sequences from the microbiome were performed as previously described (Ren et al., 2017). CD-Hit (Fu et al., 2012) was employed to cluster (95% consistency and 85% coverage) the identified viral sequences, in which the longest gene in each group was selected as the representative sequence, so as to obtain 27,079 DNA non-redundant viral scaffolds (NR_Scaffolds). Because acquiring high-genomes was crucial for our downstream analysis, we retained only NR_Scaffolds with a length of at least 5 kb and defined them as viral operational taxonomic units (vOTUs). CheckV divided the vOTUs into different groups according to assembly quality and completeness for downstream analysis.

### 2.4. Taxonomic annotation of vOTUs and their relative abundance

The vOTUs were clustered with viral sequences from the IMG/VR v 3.0 database (BLASTn; *E* value ≤10^−5^, nucleotide similarity ≥95%, and covered length ≥ 85%) to be assigned to a known viral taxonomic species. Referring to Nayfach’s research, we clustered again the vOTUs and the IMG/VR database virus sequences using a combination of gene sharing and average amino acid identity (AAI), and determined the taxonomic information of vOTUs at the genus and family levels (Nayfach et al., 2021). Besides, Contig Annotation Tool (CAT) v5.0 (von Meijenfeldt et al., 2019) was also used to taxonomically classify the vOTUs.

To calculate the relative abundance of vOTUs, metagenomic reads of all samples were mapped to the vOTU scaffolds, using Bowtie 2 with default settings (Langmead and Salzberg, 2012). The abundance of each vOTU was calculated by adopting the following equation:


$$\mathrm{Abundance}\;\left(\mathrm{coverage,}\times /\mathrm{Gb}\right)=\frac{N_{\mathrm{mapped reads}}\times L_{\mathrm{reads}}}{L_{\mathrm{vOTU sequences}}\times s}$$

where **N**_mapped reads_ is the number of mapped reads for each vOTU sequence; **L**_reads_ is the sequence length of the metagenomic reads (150 bp in this study); **L**_vOTU sequences_ is the sequence length of the vOTU; *s* is the size of each metagenomic data set (Gb).

### 2.5. Forecast the lifestyles of the bacteriophages

In specific, the PhaMer software was used to predict the sequences of bacteriophages within the vOTUs identified above (Shang et al., 2022). Then, the phage with a temperate or lytic phage was classified by PhaTYP (Shang et al., 2023). Probability scores attained from PhaTYP were between −1 and 1, which was represent the probability of “Lytic” or “Temperate.”

### 2.6. Host prediction

We used three different methods to predict host-virus connections. (1) Blastn: the vOTUs were aligned with the collected fecal samples metagenomic assembled scaffolds with the following parameters: bitscore ≥50, *e*-value ≥10^−3^, identity ≥70%, and matching length ≥ 2,500 bp (Emerson et al., 2018), (2) spacer hit: the CRISPR-Cas spacer in the metagenomic sequencing data was predicted using the PILER-CR, ignoring CRISPR-Cas with less than three spacers. BLASTn was used to compare the retain spacers with the vOTUs, and matches were selected when a maximum of one mismatch or gap over ≥95% of the spacer length (Nayfach et al., 2021), and (3) tRNA: Identification of tRNAs from the vOTUs and metagenomic assembled scaffolds with ARAGORN v1.2 (Laslett and Canback, 2004), and compared these tRNAs using BLASTn. Only hits with a 100% identity over 100% of the length were considered (Paez-Espino et al., 2016).

### 2.7. Gut virome functional annotation

The vOTUs sequence was used as queries for further functional annotation. The open reading frames (ORFs) within these vOTUs were predicted using Prodigal v2.6.3 (Hyatt et al., 2010). Then, the ORFs were used as queries to search for functional genes using BLASTp against the Kyoto Encyclopedia of Genes and Genomes (KEGG) database. The best hit comparison results of *e* < 1e-3 were screened to obtain the virus function information.

### 2.8. Network analysis

The co-correlation between the gut bacteria and viruses was constructed by calculating each pairwise Pearson’s rank correlation. Only the bacterial genera and viruses with a strong relation (|*ρ*| > 0.7 and *p* < 0.01) were considered. The network layout was visualized using Cytoscape v3. 7. 1 (Shannon et al., 2003).

### 2.9. Statistical analysis

The behavior results (body weight, SPT, LDT, and FST) were compared between groups using one-way analysis of variance (ANOVA). To evaluate the α-diversity of these fecal samples, we calculated the vOTUs diversity using the Shannon index and richness using the Sobs index. Nonmetric multidimensional scaling analysis (NMDS) based on Bray–Curtis distance was used to assess the variation of viral composition among different groups. Kruskal–Wallis test was performed to compare differences in abundance between each group, which were displayed as means ± SEM. Moreover, the differential KEGG pathway representation was identified using linear discriminant analysis effective size (LEfSe) between the two groups. The LDA score for distinguishing features was set at 2.0. Statistical analyses were conducted using the software SPSS (version 23.0, Armonk, NY, USA), and plots were generated from R and GraphPad Prism (version 8.0).

## 3. Results

### 3.1. Antidepressants and XYS improved CUMS-induced depression-like behaviors

The experimental scheme was presented in Supplementary Figure S1A, the depression-like behaviors in rats were assessed by the sucrose preference test (SPT), open field test (OFT), and light/dark test (LDT). On week fifteen immediately post-Ami, Flu, and XYS, the body weight of the XYS group was significantly higher than that of CUMS group, whereas no weight gain was significantly observed after Ami and Flu treatment compared to the CUMS rats (Supplementary Figure S1B). After six weeks of treatment with Ami, Flu, or XYS, the Ami, Flu, and XYS rats all demonstrated a significantly increased sucrose preference ratio and rearing and crossing numbers and lowered dark time (Supplementary Figure S1C–E). Among these, the SPT test results of XYS were relatively higher than that of the other two groups, and the Flu group performed best in the OFT experiment and the LDT experiment. These results revealed that antidepressants and XYS could improve depression-like behaviors in CUMS-induced rats.

### 3.2. Overview of metagenomic analyses

This study utilized the combination of shotgun metagenomics and virus sequence recognition algorithms analysis to explore the gut virome of the fecal samples from healthy, depressed, and drug-treated rats. Each fecal sample generated about 9 Gb raw reads. After quality control, more than 97% of the raw reads were considered high-quality-filtered reads, so this sequencing dataset contented the requirements of subsequent analysis (Supplementary Table S1). Then the clean reads were assembled into longer scaffolds using SPAdes. SPAdes are well proven to perform well in *de novo* whole-genome assembled (Nurk et al., 2017). In total, 776,760 scaffolds longer than 1,000 bp were attained with an average length of 3,844 bp. Screening is another crucial process to identify viral sequences from complex scaffolds. We established two bioinformatics methods to screen and identify virus sequences as shown in Supplementary Figure S2. Altogether, in all 5,029 vOTUs were discovered and quantified. We then estimated the quality and length of each viral genome with CheckV (Nayfach et al., 2021). As illustrated in Supplementary Figure S3A, 30 (0.60%) of the vOTUs were classified as complete quality, 378 (7.52%) as high quality, and 4,621 (91.88%) as genome fragments (<90% complete). In addition, nowadays, the largest virus genome found in a human sample is about 393 kb (Zhao et al., 2022). In this study, the genome length corresponding to different completeness was shown in Supplementary Figure S3B, including nine that were ≥ 393 kb (Supplementary Table S2), which may correspond to huge viruses (Al-Shayeb et al., 2020). In particular, one virus (termed vOTU-1) was assembled with lengths of 862 kb, which is longer than the largest phage genome reported so far (735 kb; Al-Shayeb et al., 2020). These results increase our understanding of huge viruses in the gut ecosystem.

### 3.3. Gut virome diversity alterations with oral antidepressants and XYS treatment

**Figure 1:**
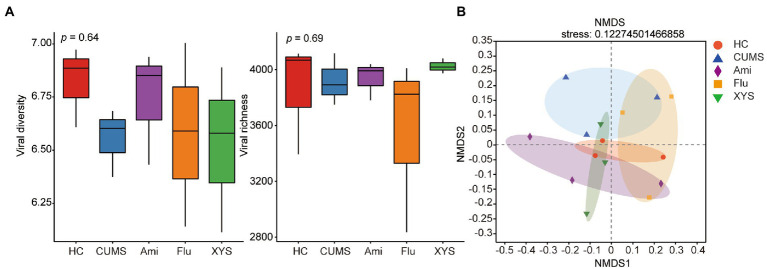
The gut virome biodiversity alterations following Ami, Flu and XYS treatment. **(A)** Comparison of viral alpha-diversity based on Shannon diversity index and Sobs richness index. **(B)** NMDS based on the Bray–Curtis distance showed distinct clusters of gut viruses in CUMS model rats from the Ami, Flu and XYS therapy, respectively. *p* values were determined by Kruskal–Wallis test. *p < 0.05, **p < 0.01, ***p < 0.001.

To assess the effect of oral antidepressants Ami, Flu, and XYS on gut viral community structure, we calculated each sample’s alpha- and beta- diversity. First, all the identified vOTUs were assessed for alpha diversity using Shannon and Sob indexes. Shannon index showed that only Ami treated mice (Ami: Shannon index = 6.741 ± 0.221 [mean ± SEM]) had reversed viral diversity when compared to CUMS mice (CMUS: Shannon index = 6.553 ± 0.131 [mean ± SEM]), and the Shannon index in Ami group was close to that in HC group (HC: Shannon index = 6.822 ± 0.155 [mean ± SEM]), due to no statistical significance between them (*p* > 0.05, Figure 1A). Besides, a recovered trend was detected for the viral richness in Ami (Ami: Sobs index = 3962.667 ± 112.036 [mean ± SEM]) and XYS (XYS: Sobs index = 4,024 ± 43.012 [mean ± SEM]) groups compared to that in CUMS (CUMS: Sobs index = 3918.667 ± 151.099 [mean ± SEM]) rats without treatment, but no significant difference was noticed (*p* > 0.05, Figure 1A).

We then compared β-diversity at the vOTUs level using the Bray–Curtis metric. NMDS demonstrated that the five groups clustered distinctly with a stress value of 0.12 (the value of less than 0.2 indicates the validity of NMDS) (Figure 1B). The NMDS results suggested that the fecal virome of the CUMS rats displayed a distinct composition compared with HC rats (Figure 1B). Intriguingly, with Ami, Flu, and XYS treatment, the viral composition of rats was also separated from CUMS rats, as assessed by NMDS analysis (Figure 1B).

### 3.4. Identification of gut viral community after oral antidepressants and XYS

**Figure 2:**
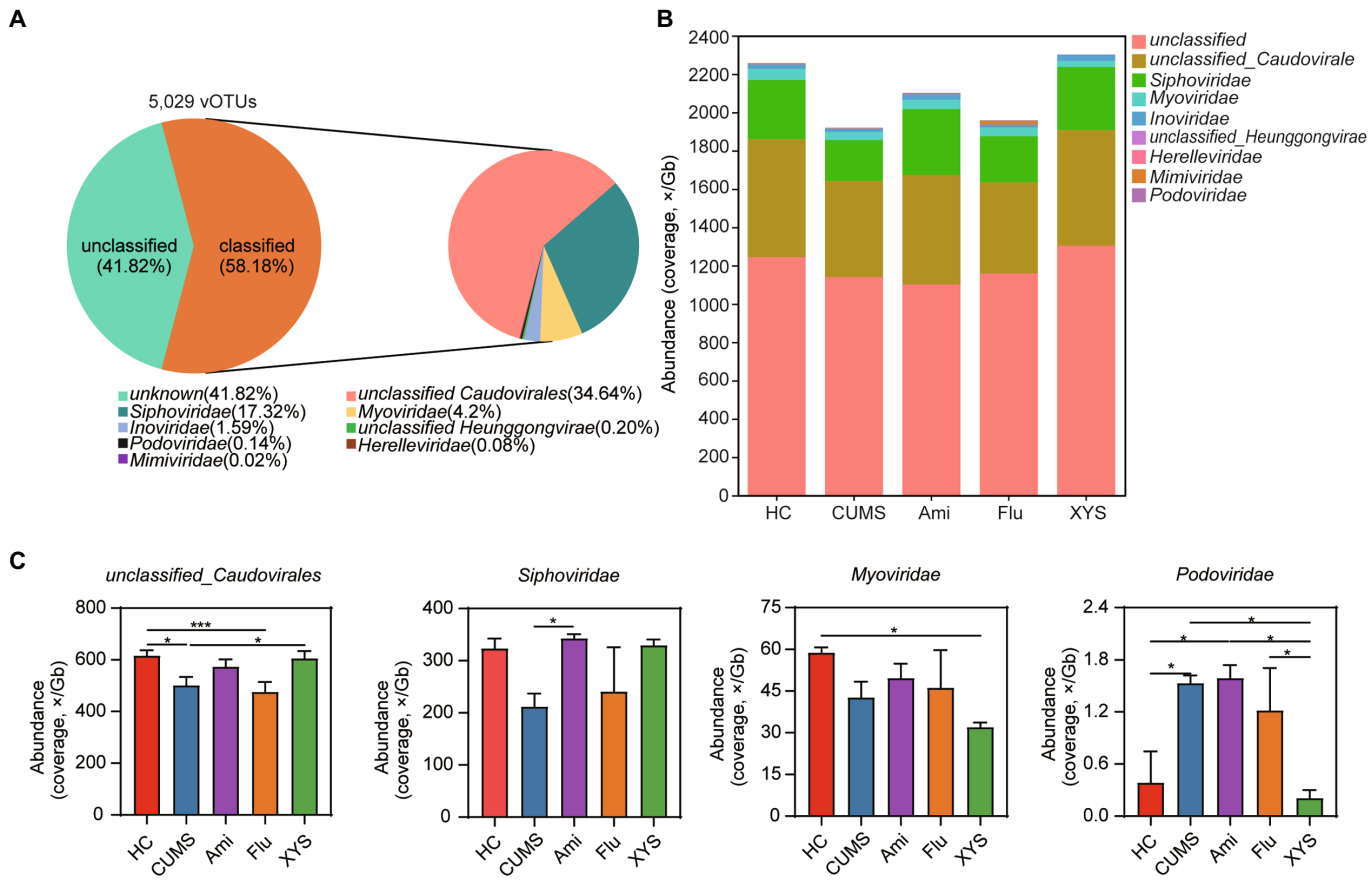
The DNA viral composition and taxa at the family levels among the five groups. **(A)** Community pie-chart showing the proportion of viral operational taxonomic units (vOTUs) that were assigned to viral taxa at the family level. **(B)** Bar plot showing the gut viral abundance of all samples at the family level. **(C)** Variation analysis to identify differentially present taxa between the five groups at the family levels. Significant different are indicated: *p < 0.05, **p < 0.01, ***p < 0.001.

To further quantify the changes in gut virome after treatment with antidepressants and XYS, we attempted to classify vOTUs at the species, genus, and family levels. At the family level, 58.18% of the vOTUs were classified into eight families, which were mainly dominated by bacteriophages from the *unclassified_Caudovirales*, *Siphoviridae*, and *Myoviridae* (Figures 2A,B). Furthermore, our study also found several gut viruses in the giant virus family *Mimiviridae* (Figure 2B). At the genus level, 75.58% of the vOTUs were unclassified (Supplementary Figure S4A). No vOTUs were annotated at the species level. These results show that there was still a large proportion of unclassified viruses at the family and genus level, suggesting gut unexplored viral diversity.

Next, we performed variation analysis to distinguish differentially present taxa between the five groups at family and genus levels. As shown in Figure 2C, *unclassified_Caudovirales* was higher in the HC group than in CUMS group (Kruskal−Wallis test, *p* < 0.05), whereas *Podoviridae* (*p* < 0.05) was enriched in the CUMS group relative to the HC group. After six weeks of intervention with three antidepressant drugs, the family *unclassified_Caudovirales* was found to be increased in the Ami and XYS groups, while only the XYS group exhibited significant differences as compared to CUMS (*p* < 0.05). We also observed that XYS significantly decreased the abundance of *Podoviridae* compared to those in CUMS (*p* < 0.05). Moreover, compared to that of CUMS group, although both oral Ami and XYS could improve the abundance of *Siphoviridae*, only Ami treatment resulted in statistical significance and achieved levels alike to those of HC (*p* < 0.05). It is worth noting that the family *Myoviridae* was significantly underrepresented in the XYS group and reached a significant level with respect to HC (*p* < 0.05). At the genus level, both Ami, Flu, and XYS upregulated the abundance of *g_unclassified_Caudovirales* compared with CUMS group (Supplementary Figure S4A). However, only the Ami group reached a remarkable difference (*p* < 0.05; Supplementary Figure S4B). Compared to CUMS, only Flu treatment resulted in a lower abundance of *g_unclassified_Inoviridae* (*p* < 0.05; Supplementary Figure S4B). Ami, Flu, and XYS treatment was also associated with decreased the abundance of *Hendrixvirus*. Among these, Flu and XYS groups were significantly decreased (*p* < 0.05; Supplementary Figure S3B). Moreover, the abundance of *Phifelvirus* was enriched in Ami group relative to CUMS, Flu and XYS groups (*p* < 0.05; Supplementary Figure S4B). Then in order to identify the common and unique gut virome among the five groups, the gut viral community composition was then compared at the vOTUs level. The Venn diagram displayed that 3,926 vOTUs were shared among the five groups, while 59, 43, 53, 26, and 43 were unique to HC, CUMS, Ami, Flu, and XYS groups (Supplementary Figure S4C).

### 3.5. Functional alterations of gut virome after oral antidepressants and XYS

**Figure 3:**
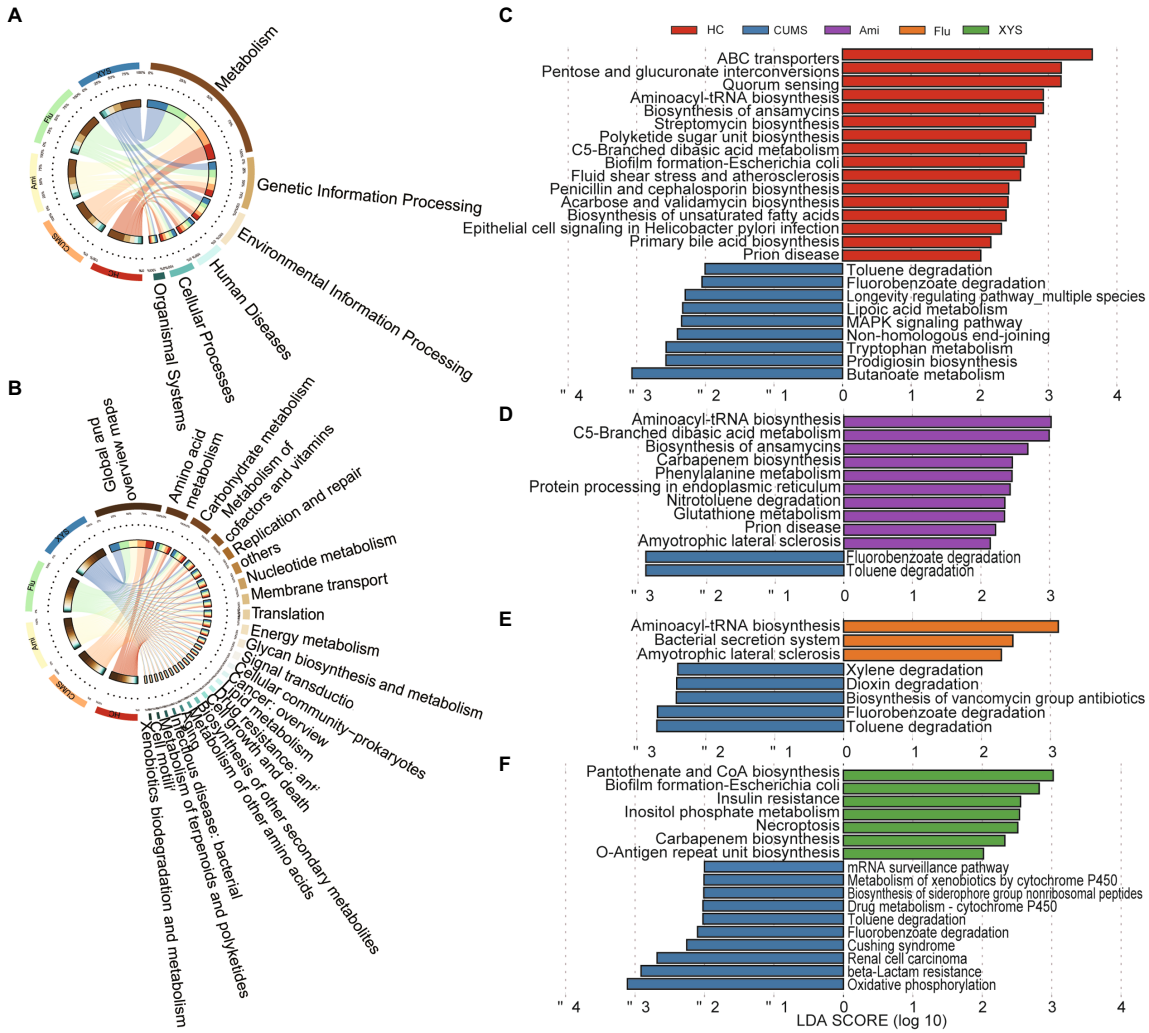
The functional profiles of gut virome in the KEGG pathway *via* metagenomics sequencing. Distribution of KEGG pathways at **(A)** level 1 and **(B)** level 2. LEfSe analysis of the KEGG pathways with the criteria of LDA > 2.0. **(C)** Comparison between the HC and CUMS on the level 3 of KEGG pathways. **(D)** Comparison between the CUMS and Ami on the level 3 of KEGG pathways. **(E)** Comparison between the CUMS and Flu on the level 3 of KEGG pathways. **(F)** Comparison between the CUMS and XYS on the level 3 of KEGG pathways.

To outline the effects of antidepressants and XYS on the function of gut virome, we annotated the identified vOTUs set from the gut virome using the KEGG database. In the first-level KEGG pathways, the most abundant functional categories in the different groups were associated with metabolism and genetic information processing (Figure 3A). Among them, the majority of metabolism functions consisted of global and overview maps, carbohydrate and amino acid metabolism (Figure 3B). As for genetic information processing, the proportion of functional genes related to replication and repair was the highest (Figure 3B). Furthermore, we also observed that some genes were directly related to the bacterial hosts, including cellular community: prokaryotes, drug resistance: antimicrobial and infectious disease: bacterial (Figure 3B).

Additionally, to further investigate the function of the drug-related gut viruses, we applied a LEfSe analysis on the third-level KEGG pathway. As illustrated in Figure 3C, 25 different KEGG pathways were identified between HC and CUMS groups (LDA > 2.0). The 9 CUMS-enriched KEGG pathways consisted of amino acid metabolism, carbohydrate metabolism, xenobiotics biodegradation, and metabolism, metabolism of cofactors and vitamins, biosynthesis of other secondary metabolites, replication and repair, aging, and signal transduction. Compared with CUMS group, Ami, Flu, and XYS treatment resulted in 10, 3, and 7 KEGG pathways differences, respectively (Figures 3D–F). Notably, pathway related to xenobiotics biodegradation and metabolism (toluene degradation and fluorobenzoate degradation) was decreased in Ami, Flu, and XYS treatment group, in line with the decreased functions in the HC group (Figures 3D–F). In contrast, pathways of cellular community-prokaryotes (biofilm formation-*Escherichia coli*) and translation (aminoacyl-tRNA biosynthesis) were enriched in the XYS and Flu groups, separately, in agreement with the enriched functions in the HC group (Figures 3E,F). Moreover, after Ami intervention for six weeks, we also found that pathways associated with translation (aminoacyl-tRNA biosynthesis), carbohydrate metabolism (c5-branched dibasic acid metabolism), and neurodegenerative disease (prion disease) were increased in Ami group, consistent with the increased function in the HC group (Figure 3D).

### 3.6. Predicting the lifestyle and host of viruses and co-occurrence network analysis between viruses and their bacterial host

**Figure 4:**
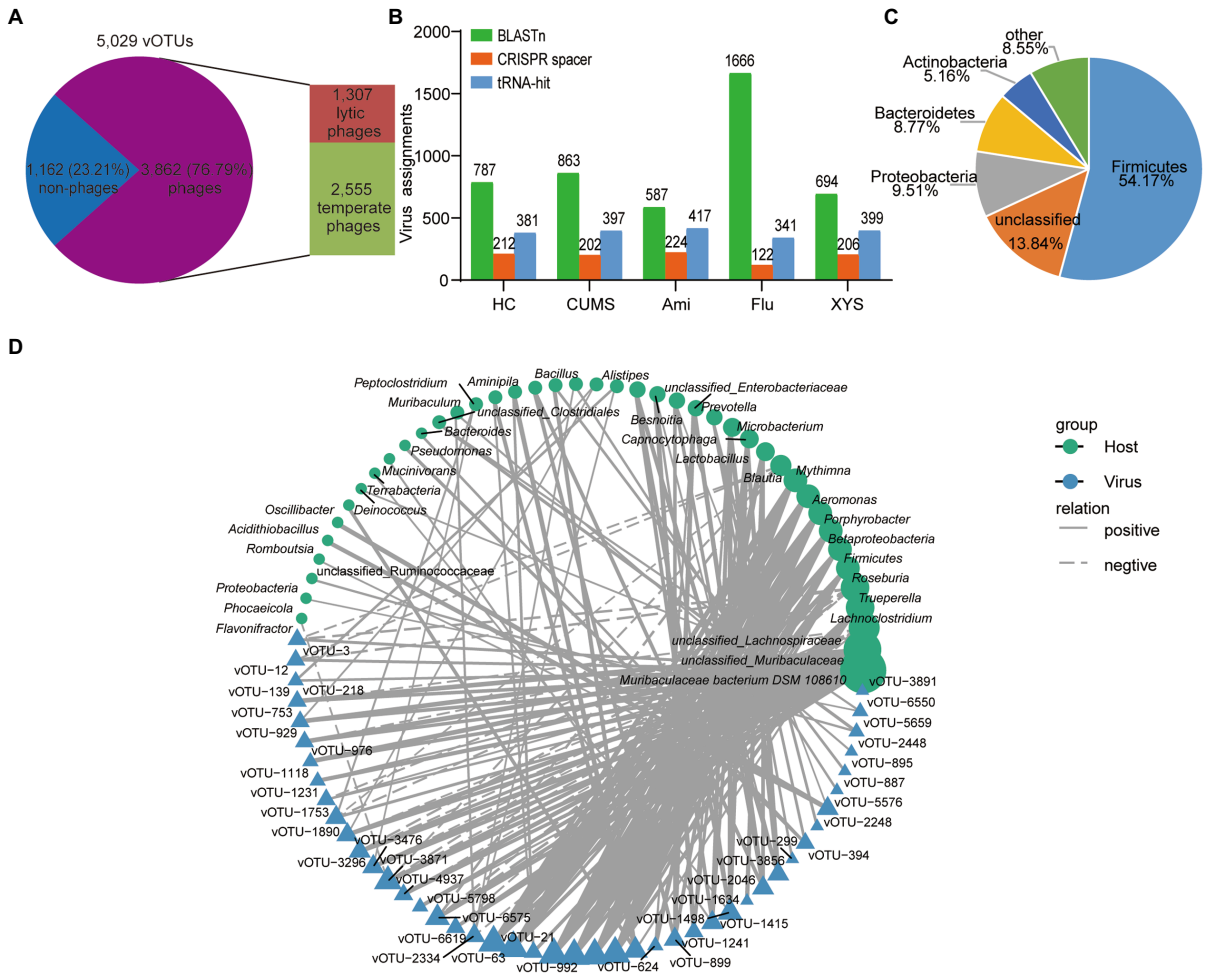
Correlations between gut viruses and bacterial hosts. **(A)** The distribution of the temperate and lytic phages. **(B)** Total number of host assignments to metagenomic viruses using three methods. Total assignments for BLASTn, CRISPR spacer and tRNA-hit are shown above each bar. **(C)** The proportion of vOTUs that are predicted to infect microbial hosts at the phylum level. **(D)** The interaction network of bacterial genera and vOTUs determined by Cytoscape v3.7.1. Only connections with a strong (Pearson’s |*ρ*| > 0.7) and significant (*p* < 0.01) correlation are presented in the network. Circles and triangles represented the different bacteria and viruses, with solid and dashed lines indicating the positive and negative correlations, respectively.

The interaction between gut viruses and their bacterial hosts dominates the intestinal ecosystem. The gut virome was mainly composed of bacteriophages (phages). Predicting the life cycles of phage could contribute to deciphering their interactions with bacterial hosts. Toward this goal, we assessed the percentage of lytic or temperate phages in the vOTUs. In all, 3,862 phage vOTUs were identified, including 2,555 (66.16%) temperate phages and 1,307 (33.84%) lytic phages (Figure 4A). Notably, temperate phages were the predominant viruses, accounting for more than 65% of the phages’ vOTUs.

Then, we sought to identify the bacterial hosts of the 5,029 vOTUs using three different bioinformatics methods to present how these viruses affect the health of organisms by infecting microbial hosts. First, we testified that 4.2% (212/5,029), 4.0% (202/5,029), 4.5% (224/5,029), 2.4% (122/5,029), and 4.1% (206/5,029) of viruses in the five groups of the gut ecosystem could be connected to a host when using CRISPR spacers strategy, respectively (Figure 4B; Supplementary Table S3). These results indicated considerable CRISPR diversity between bacterial hosts and active infection. Among the spacers in CRISPR arrays record the history of gut viruses infecting bacterial hosts, although most viruses were targeted by the spacer, CRISPR arrays were discovered in only <0.01% of metagenomic scaffolds in each group (Supplementary Table S6), affirming the limited distribution of this anti-viral defense system (Burstein et al., 2016). Then, we were able to assign 381, 397, 417, 341, and 399 viral sequences to bacterial hosts using tRNA in each group (Figure 4B; Supplementary Table S4). Furthermore, to expand the host-virus network, we identified connections between the viral sequences and metagenomic scaffolds in each group based on direct BLASTn matches (bitscore ≥50, *e*-value ≥10^−3^, identity ≥70%, and matching length ≥ 2,500 bp), resulting in the connection to the bacterial hosts that more than 10% of viral sequences in each group (Figure 4B; Supplementary Table S5). Among them, we were able to identify 123 consistent viral-host links using two or more methods (Supplement Table S7). Overall, these methods identified 25,106 putative host-virus links enabling host assignment to 48.92% (2,460/5,029) of the vOTUs (Supplementary Table S7). Among these vOTUs, a total of 59.15% (1,455/2460) of the vOTUs could be assigned to multiple microbial hosts (Supplementary Table S7). Most predicted hosts were Firmicutes, Proteobacteria, Bacteroidetes, and Actinobacteria (Figure 4C).

In addition, the gut’s relationship between virome and bacteriome is the core of intestine microbiota balance, but the interaction between viruses and bacteria has yet to be well defined. To further link the correlation, the most abundant 50 vOTUs and their bacterial hosts were visualized based on Pearson’s rank correlation (|*ρ*| > 0.7, *p* < 0.01) correlations (Figure 4D). Supplementary Table S8 summarizes the detailed co-occurrence between vOTUs and their bacterial hosts. As shown in Figure 4D, the co-occurrence networks revealed that 42 bacterial genera might serve as the potential hosts for the 45 vOTUs. In which 45 vOTUs, 84% (38/45) viruses were linked to more than one bacterial host, indicating that gut viruses also produce cascading effects (Hsu et al., 2019). Of these vOTUs were mainly assigned to *unclassified_Caudovirales*, *Siphoviridae*, and *Inoviridae* families (Supplementary Table S8). Among these 42 genera, 71% (30/42) bacterial genera could be hosts of different viruses, most of which belonged to Firmicutes, Bacteroidota, Proteobacteria, and Actinobacteria phyla (Figure 4D). Interestingly, *Muribaculaceae bacterium DSM 108610*, belonging to Bacteroidetes, carried more diverse viruses than other genera, indicating that they are the essential microbes dominating the gut ecosystem. In a word, these results suggested that the potential role of these viruses in regulating the bacterial communities in the gut.

## 4. Discussions

Depression is a common mental disorder with a high global incidence. Drugs represent a primary and extensive application for the treatment of depression (Cipriani et al., 2018). There is abundant evidence that drugs have been proven to impact the composition and function of the intestinal microbiota and can be seen as a vital “organ” for maintaining host health (Zhu et al., 2019; Duan et al., 2021; Li et al., 2021). In our previous study, we reported that the treatment of Ami and XYS all lead to an increase in microbial alpha-diversity and relative abundance of healthy gut bacteria, such as *Bacteroides*, and Ami and Flu treatments may also increase relative abundance of microbial subpopulation with negative effects on health, such as Porphyromonadaceae and *Alistipes* (Qu et al., 2019; Zhang et al., 2021). However, the gut also has been shown to include a diverse virome (Shkoporov and Hill, 2019). Several recent studies have found that the treatment of drugs may also help to reconstruct and restore the virome signature in the disease group (Seth et al., 2019; Yang J. et al., 2020). Thus, the above study prompted us to explore the impacts of antidepressant drugs on the gut virome as a possible therapeutic target.

To our knowledge, this is the first time to survey the impact of Ami, Flu, and XYS on the structure and function of the intestinal virome in rats exposed to chronic unpredictable mild stress. In this study, we found the imbalance of virus community, taxonomic shifts, and functional changes between CUMS and HC rats, and after administration of Ami, Flu, and XYS might influence gut virome profile and function in CUMS-induced rats. Furthermore, through various methods, we could predict host-virus associations, which may be crucial for future understanding of the pathogenesis of depression, designing phage therapy, or comprehending the co-evolutionary dynamics of host and virus. Overall, these data reinforce the previous bacteriome study on drug-treated depression (Qu et al., 2019; Zhang et al., 2021) by increasing new information about the virome.

The alpha index of the gut virome of CUMS-induced rats led to a decreasing tendency compared with HC rats. After the intervention of three different drugs, the diversity index was increased in Ami and XYS groups, whereas this trend was not observed in the Flu group, although there were no statistical differences in alpha diversity of the gut virome among the five groups. These results are consistent with a similar trend in bacterial richness and diversity observed in our published research, suggesting the alterations in viral diversity might be linked to the changes in the diversity of intestinal flora (Qu et al., 2019; Zhang et al., 2021). Similar to our results, Yang et al. demonstrated a significant reduction in gut viral alpha diversity in major depressive disorder patients relative to healthy subjects (Yang H. J. et al., 2020). Furthermore, a reduction of gut viral diversity has also been discovered in patients with other diseases, such as irritable bowel syndrome (Coughlan et al., 2021), polycystic ovary syndrome (Huang et al., 2022), Parkinson (Tetz et al., 2018), and so on. However, data on the latent correlations between illness and gut virome alpha diversity are inconsistent. For example, Yang et al. also detected a rise in gut viral alpha diversity in obesity and type 2 diabetes mellitus cohort (Yang et al., 2021), but a previous study reported that there were no statistically significant between hypertension and healthy subjects in alpha diversity (Han et al., 2018).

Viruses are divided into 233 families based on the latest (2021) report by the International Committee for the Taxonomy of Viruses (ICTV, Virus Metadata Repository: version March 2021; MSL37). In the human gut and many ecosystems, the gut viral communities display highly diverse, stable, and personalized and are dominated by Caudovirales phages (Shkoporov and Hill, 2019). Furthermore, although the genome of viruses could be DNA or RNA, we have paid attention to the abundant DNA virome in our research. Here, our results showed that the gut DNA virome was mainly dominated by *f_unclassified_Caudovirales*, *Siphoviridae*, *Myoviridae*, and *Podoviridae*, which was in accordance with previous studies (Norman et al., 2015; Ma et al., 2018; Zuo et al., 2019). Among these, *Siphoviridae*, the most abundant virus family of the normal human intestinal virus community, was discovered to be less distributed in the gut virome of CUMS-induced rats than in the HC rats, while the abundance of *Siphoviridae* significantly restored after Ami treatment. *Siphoviridae* is a double-stranded (ds) DNA virus and infects a variety of intestinal bacteria, such as *Bacillus*, *Lactobacillus*, *Enterococcus*, *Streptococcus*, and so on (Adriaenssens et al., 2015). Our results are consistent with the previous human study that reported increased *Caudovirales* and *Siphoviridae* levels exhibited could improve functioning in verbal memory and executive processes (Mayneris-Perxachs et al., 2022). In addition, our result found that increased levels of *Podoviridae* in CUMS rats, while the profile was then significantly decreased after XYS treatment. Consistent with our findings, some studies have shown that *Podoviridae* was richer in ulcerative colitis than in controls and may increase the severity of the disease, although the mechanism remains unknown (Zuo et al., 2019). Interestingly, we also found the *Mimiviridae* family of giant viruses in the gut (Figure 2B), which may be associated with depression. However, the clinical significance of the presence of giant viruses in the intestine is still unknown. They may be transient travelers or infect intestinal cells, encountering translocation to other parts of the body or contributing to local inflammation (Colson et al., 2013). Nonetheless, we were still unable to identify ssDNA bacteriophages (mostly *Microviridae* family members) in the gut DNA virome, which may be due to inadequate nucleic acid extraction procedures.

Based on the KEGG database, we further revealed the functional alterations of gut virome following Ami, Flu, and XYS treatment, respectively. Firstly, in our study, the central gut virus-associated functional genes were divided into metabolism and genetic information processing, which aligns with the results from our previous study that gut bacteriome was also enriched in metabolism and genetic information processing (Zhang et al., 2021). Secondly, we also discovered there were several functions associated with the bacterial hosts, including prokaryote cellular community, antimicrobial drug resistance, and bacterial infectious disease categories, suggesting that gut viruses have host-regulating functions in the gut. This resembled that of the mucosal virome in ulcerative colitis patients, which also possess dominant viral functions associated with bacteria fitness, pathogenicity, and antibiotic resistance (Zuo et al., 2019). Last but not least, our result illustrated that CUMS rats were characterized by clear enrichment of xenobiotics biodegradation and metabolism, whereas the Ami, Flu, and XYS treated rats all led to a decrease in this function. This result implied that depressed rats might have higher energy demand, and after the treatment of Ami, Flu, and XYS alter the gut virome metabolic functions under conditions of depression. In brief, these data support that the treatment of Ami, Flu, and XYS potentially has supplemental influences beyond anti-depression due to its effect on the function of the gut virome.

Additionally, predicting the bacterial hosts of viruses is critical to understand the potential role of viruses in the development of depression and a necessary first step to designing innovative phage therapies using host-virus interactions (Lin et al., 2017). Based on the objectives, we used three different bioinformatics methods to study how these viruses affect the health of organisms by infecting microbial hosts. The result showed that BLASTn matched the greatest number of assigned viruses, followed by tRNAs and CRISPR spacers. Here we also conducted the most generalized analysis of the host range of enteroviruses. The result showed that a significant percentage (more than 80%) was expected to infect multiple bacterial genera. The wide range of host-range viruses is due to enhanced connectivity of horizontal gene transfer events, which may lead to the establishment of gene-flow networks between different phylogenetic bacterial species in the intestine. Furthermore, among the predicted microbial hosts, Firmicutes, Proteobacteria, Bacteroidetes, and Actinobacteria were the dominant bacteria. These findings were consistent with previous reports on the predominant microbiota in the human intestine (Zoetendal et al., 2008; de Vos et al., 2022). It is interesting to note that many viruses are related to the genus of Bacteroides, reflecting the relationships between Bacteroides and viruses. These findings indicate that host-virus interactions can be systematically illustrated by analyzing viral and microbial genomes from the same environment.

In the last part of our study, correlation analysis of the most abundant viruses and bacteria genera showed that an extensive positive correlation between viruses and bacteria existed in the gut ecosystem (Figure 4C), which is in contrast to previous studies of viruses in the intestinal mucosa of ulcerative colitis (Zuo et al., 2019). However, the mechanism is still unclear. We guessed that this result was obtained from the dynamic relationship between bacteria and bacteriophages dominant in viruses. To this day, two primaries bacteriome-phageome dynamics have been identified that explain the dynamic alterations that take place in phage-bacteria co-evolution. Among them, one is the “kill the winner” model, in which phages are known to be major predators of gut bacteria (Winter et al., 2010). The other is the “piggyback-the-winner” model, whereby intestinal phages are mainly controlled by lysogenic phages, leading to a stable virus to microbe ratio (Silveira and Rohwer, 2016). Our results are consistent with the latter model, indicating that most of the gut phages in our study may keep lysogenic or lysogenic interactions with their bacteria hosts. Lysogenic phages could positively change the physiological properties of host bacteria (Brüssow et al., 2004). To verify the above assumptions, we also investigated the life cycles of the identified phages in the gut virome. Consistent with the previous study, temperate phages were the predominant phages in the gut ecosystem (Coughlan et al., 2021), and might make a significant contribution to defending the host from pathogen colonization (Manrique et al., 2017). The further study requires to explore the balance between lytic and temperate phages and their potential health influences.

Some limitations in our study include (i) we have not sequenced the RNA virome, which is increasingly considered to be a crucial part of the intestinal microbiome related to health and disease (Virgin, 2014); (ii) the pathophysiology of patients with depression cannot be reproduced entirely using animal model, and the hypothesis of this study needs to be verified by more clinical data.

In conclusion, our results emphasize the importance of gut virome in depression with characterized alters in community diversity, taxon abundance, and function profiles. Also, we found that the administration of Ami, Flu, and XYS might affect gut virome profile and function in CUMS-induced rats. These results indicate that we should pay more attention to the alterations of gut viruses in depression, and the feasibility of Ami, Flu, and XYS treatment in depression by the modulating gut viral community. However, research on gut virome is still preliminary, and further clinical studies are needed to confirm the causality relationship of the gut virome in the drug therapeutics of depression.

## Data availability statement

The datasets presented in this study can be found in online repositories. The names of the repository/repositories and accession number(s) can be found in the article/Supplementary material.

## Ethics statement

The animal study was reviewed and approved by Institutional Animal Care and the Committee on the Ethics of Animal Experiments of South China Agricultural University.

## Author contributions

JL analysed the high-throughput sequencing data, wrote and revised the manuscript. WQ performed the experiments. CH modified the language. ZL gave valuable input on data analysis and revised the manuscript. HY designed and supervised the study, received the grants, reviewed the results, and reviewed and revised the manuscript. All authors contributed to the article and approved the submitted version.

## Funding

This work was supported by the National Natural Science Foundation of China (grant no. 32170064).

## Conflict of interest

The authors declare that the research was conducted in the absence of any commercial or financial relationships that could be construed as a potential conflict of interest.

## Publisher’s note

All claims expressed in this article are solely those of the authors and do not necessarily represent those of their affiliated organizations, or those of the publisher, the editors and the reviewers. Any product that may be evaluated in this article, or claim that may be made by its manufacturer, is not guaranteed or endorsed by the publisher.

## Footnotes

^1^ https://github.com/najoshi/sickle

^2^ http://bio-bwa.sourceforge.net

^3^ https://www.protocols.io/view/viral-sequence-identification-sop-with-virsorter2-5qpvoyqebg4o/v3

## Supplementary material

SUPPLEMENTARY TABLE S1 Raw data and clean data after quality control.

SUPPLEMENTARY TABLE S2 CheckV assesses the quality and completeness of metagenome-assembled viral genomes.

SUPPLEMENTARY TABLE S3 CRISPR-Cas spacer in our metagenomic scaffolds matches from the viral sequences in each group.

SUPPLEMENTARY TABLE S4 Host assignment of isolate viruses based on viral tRNAs in each group.

SUPPLEMENTARY TABLE S5 Microbial hosts connected to viral sequences based on direct BLASTn matches in each group.

SUPPLEMENTARY TABLE S6 List of bacterial hosts connected with viral sequences.

SUPPLEMENTARY TABLE S7 List of host assignment per vOTUs. Host assignment for vOTUs was determined using Blastn, CRISPRs, and tRNAs matching.

SUPPLEMENTARY TABLE S8 Strong (|ρ| > 0.7) and significant (*p* < 0.01) correlations between vOTUs and bacterial hosts.

SUPPLEMENTARY FIGURE S1 Specific flow chart of experimental design **(A)** and Antidepressants and XYS ameliorated depression-like behaviors on CUMS-induced depression-like mice **(B)**. **p* < 0.05, ***p* < 0.01, ****p* < 0.001 (compared with the HC group), #*p* < 0.05, ##*p* < 0.01, ###*p* < 0.001 (compared with the CUMS group).

SUPPLEMENTARY FIGURE S2 The two bioinformatics methods to screen and identify virus sequences is shown.

SUPPLEMENTARY FIGURE S3 The quality **(A)** and length **(B)** of each viral genome was assessed with CheckV.

SUPPLEMENTARY FIGURE S4 The DNA viral composition and taxa at the genus levels among the five groups. **(A)** Community pie-chart showing the proportion of viral operational taxonomic units (vOTUs) that were assigned to viral taxa at the genus level. **(B)** Bar plot showing the gut viral abundance of all samples at the genus level. **(C)** The Venn diagram showed that the common and unique gut virome among the five groups.
