# Supplementary figures and images for: Antidepressants amitriptyline, fluoxetine, and traditional Chinese medicine Xiaoyaosan caused alterations in gut DNA virome composition and function in rats exposed chronic unpredictable mild stress
Source: Front Microbiol. 2023 Apr 14;14:1132403. doi: 10.3389/fmicb.2023.1132403 (PMC10140408; doi:10.3389/fmicb.2023.1132403)

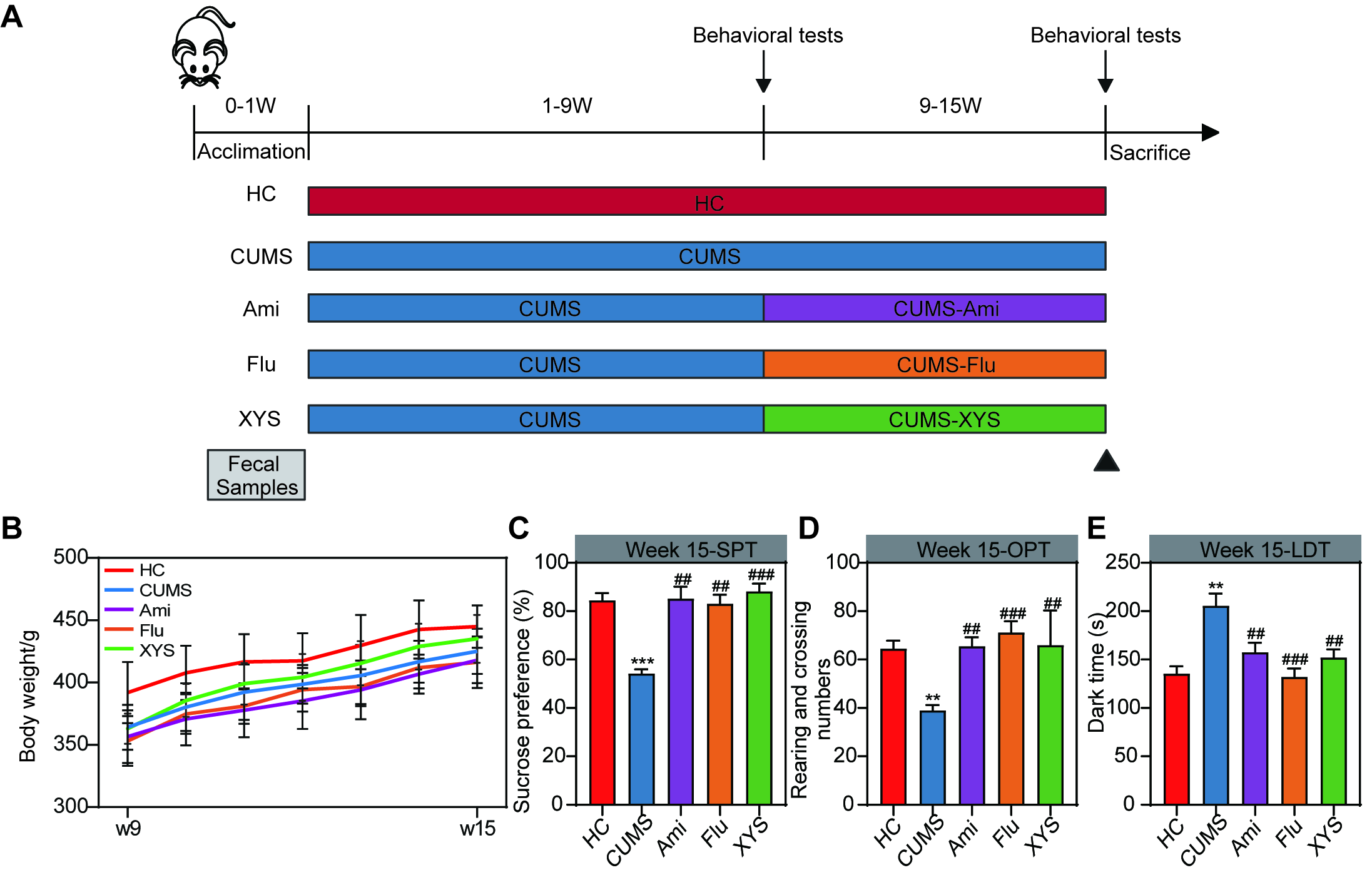

Supplement: SUPPLEMENTARY FIGURE S1 — Specific flow chart of experimental design (A) and Antidepressants and XYS ameliorated depression-like behaviors on CUMS-induced depression-like mice (B). *p < 0.05, **p < 0.01, ***p < 0.001 (compared with the HC group), #p < 0.05, ##p < 0.01, ###p < 0.001 (compared with the CUMS group). [file Image_1.tif]

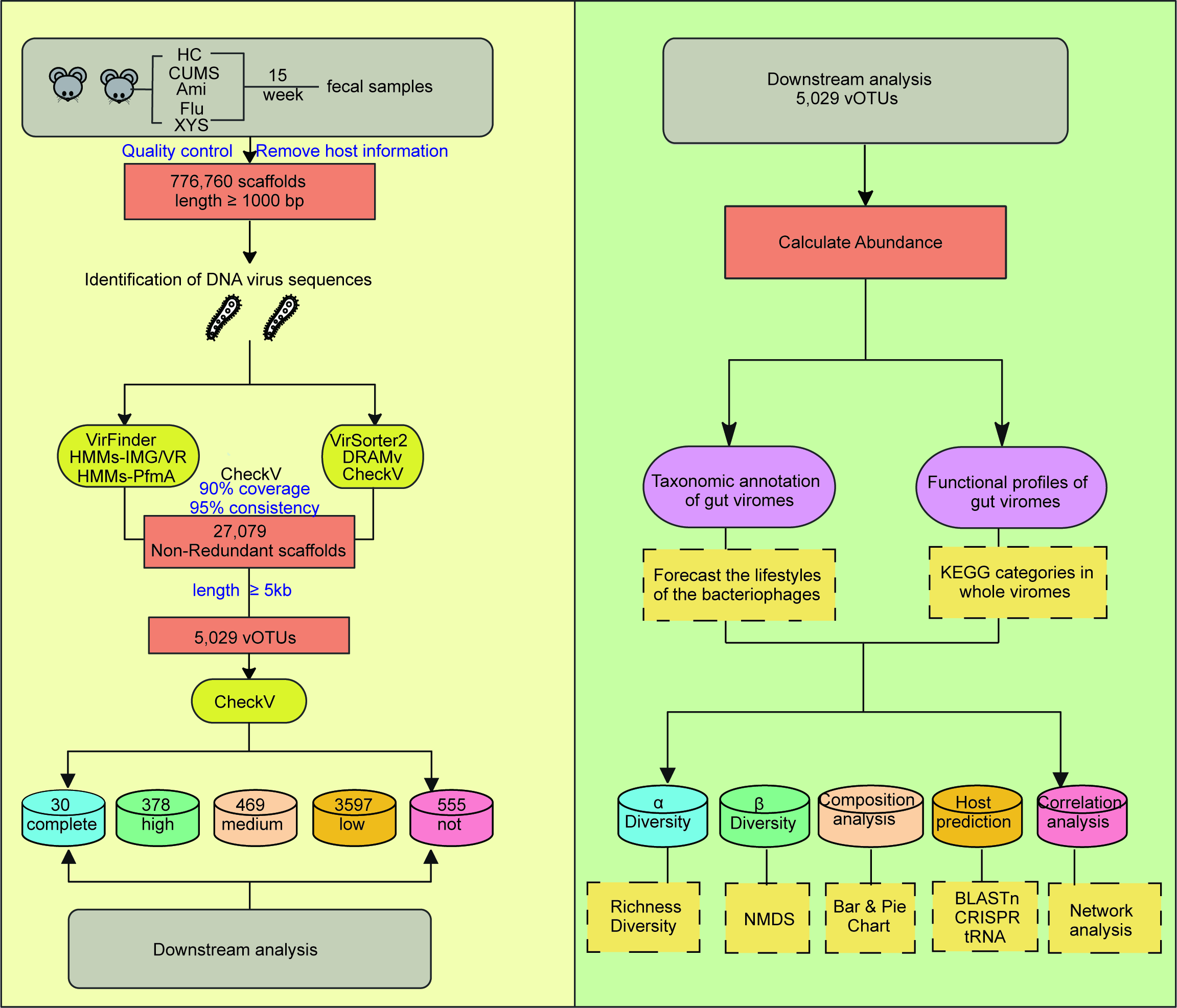

Supplement: SUPPLEMENTARY FIGURE S2 — The two bioinformatics methods to screen and identify virus sequences is shown. [file Image_2.tif]

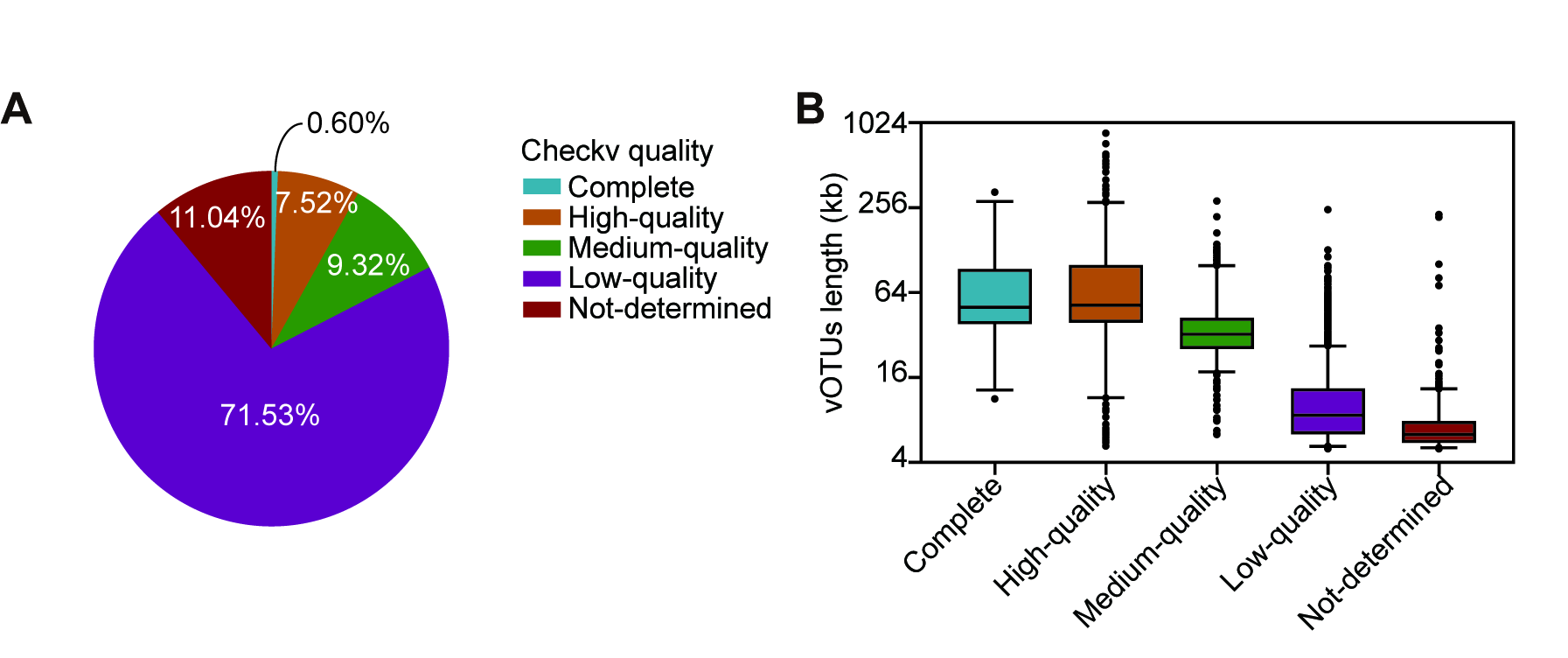

Supplement: SUPPLEMENTARY FIGURE S3 — The quality (A) and length (B) of each viral genome was assessed with CheckV. [file Image_3.tif]

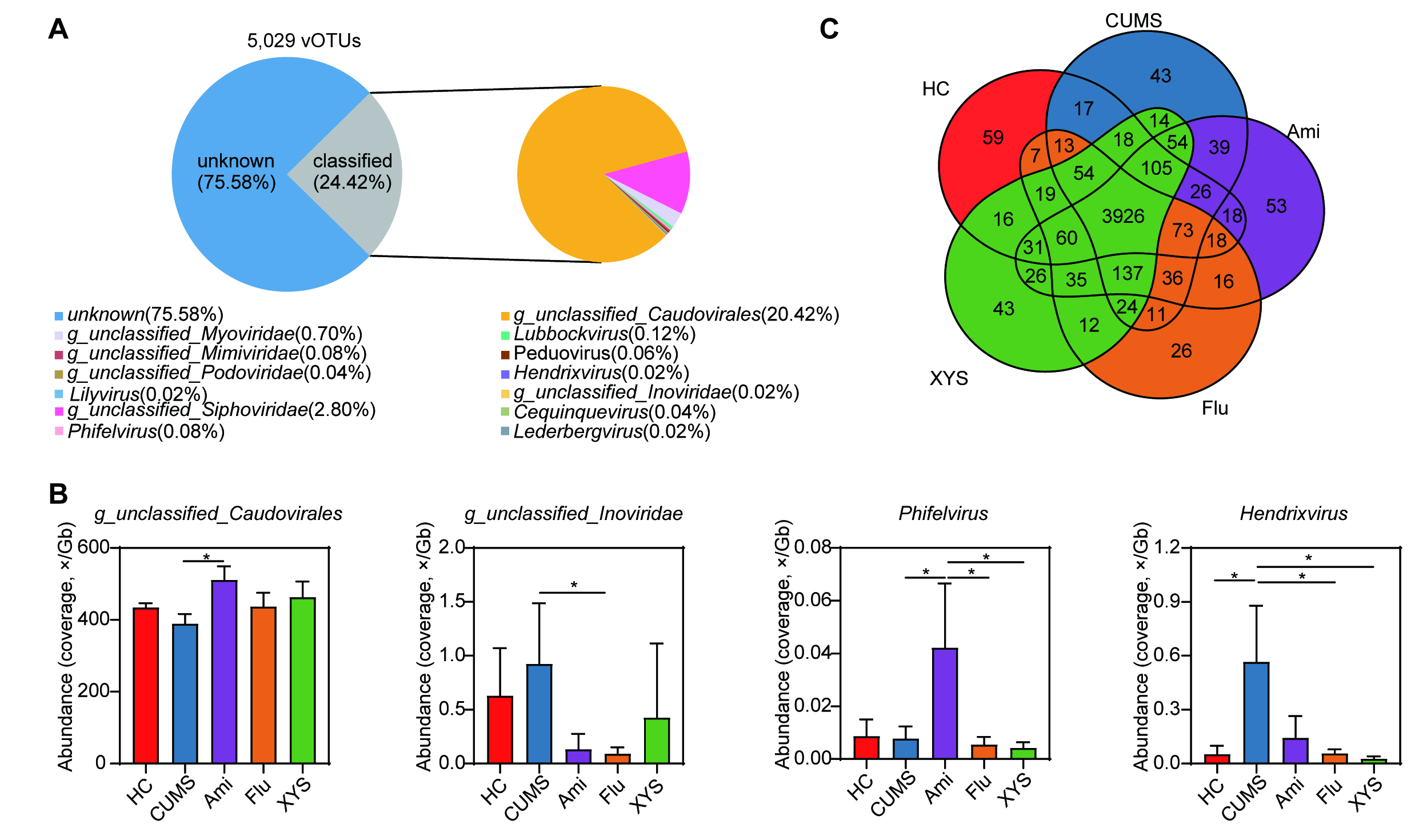

Supplement: SUPPLEMENTARY FIGURE S4 — The DNA viral composition and taxa at the genus levels among the five groups. (A) Community pie-chart showing the proportion of viral operational taxonomic units (vOTUs) that were assigned to viral taxa at the genus level. (B) Bar plot showing the gut viral abundance of all samples at the genus level. (C) The Venn diagram showed that the common and unique gut virome among the five groups. [file Image_4.tif]
